# Effectiveness of Mind–Body Exercise in Older Adults With Sarcopenia and Frailty: A Systematic Review and Meta‐Analysis

**DOI:** 10.1002/jcsm.13806

**Published:** 2025-04-20

**Authors:** Ruihan Wan, Jie Huang, Kangle Wang, Danting Long, Aolong Tao, Jia Huang, Zhizhen Liu

**Affiliations:** ^1^ College of Rehabilitation Medicine Fujian University of Traditional Chinese Medicine Fuzhou Fujian China; ^2^ National‐Local Joint Engineering Research Center of Rehabilitation Medicine Technology Fujian University of Traditional Chinese Medicine Fuzhou Fujian China

**Keywords:** meta‐analysis, mind–body exercise, older adults, sarcopenia and frailty

## Abstract

**Background:**

Mind–body exercise (MBE) has shown promise in mitigating the effects of sarcopenia and frailty in older adults. Nevertheless, its effectiveness in enhancing muscle function and physical performance in this population has not been well established. This study aimed to investigate the effects of MBE on older adults with sarcopenia and frailty, to offer evidence‐based exercise recommendations.

**Methods:**

A comprehensive search for randomized controlled trials (RCTs) was conducted through multiple databases, including PubMed, Embase, Cochrane Library, Web of Science, PsycINFO, CINAHL, China National Knowledge Infrastructure (CNKI), WanFang, and Chinese Scientific Journals Full‐Text Database (VIP), supplemented by manual reference searches from inception until February 2024. The eligible RCTs compared MBE with passive or active exercise controls, focusing on muscle function and physical performance in older adults aged 60 years or above. Subgroup analyses were conducted to evaluate the types, duration, and frequency of MBE.

**Results:**

Nine eligible RCTs with 1838 participants were included in this study. MBE demonstrated significant improvements compared with passive control, particularly in grip strength (WMD [weighted mean difference] = 0.99; 95% CI [95% confidence interval] = 0.06, 1.92; *I* ^2^ = 3%, *p* = 0.04), Timed Up and Go Test (TUGT) (WMD = −4.04; 95% CI = −5.54, −2.53; *I* ^2^ = 12%, *p* < 0.01), and Berg Balance Scale (BBS) scores (WMD = 3.63; 95% CI = 0.38, 6.87; *I* ^2^ = 0%, *p* = 0.03). Even when compared to active exercise training, improvements were still observed in TUGT and BBS (*p* < 0.001), with a trend toward improved grip strength (WMD = −2.20; 95% CI = −4.35, −0.04; *p* = 0.05). No positive effect on muscle mass was observed. Subgroup analysis indicated that MBE performed more than 5 times a week for a short or medium duration (4–24 weeks) could improve grip strength (*p* < 0.05). Moderate‐frequency intervention over a short period in this population yielded greater improvements in gait speed and Chair Rise Test completion time (*p* < 0.05).

**Conclusions:**

MBE can enhance muscle function and physical performance to some extent in older adults with sarcopenia and frailty, whether they are compared with passive or active exercise training. However, positive effects on muscle mass have not been observed. Future studies are warranted to compare it with well‐designed active exercise training programs that match the exercise volume, to draw more definitive conclusions to support the notion that MBE yields comparable effects.

## 1 Introduction

With the accelerated pace of ageing of the global population, sarcopenia and frailty have emerged as prominent health concerns among older adults as a geriatric syndrome associated with ageing [1, 2]. Both conditions have similar clinical manifestations, including muscle atrophy and diminished function [3, 4]. Sarcopenia, which is a progressive form of skeletal muscle degeneration, typically precedes frailty, representing a more severe stage characterized by reduced physical activity and low body weight [5, 6]. Evidence from a systematic review and meta‐analysis indicates that sarcopenia and frailty generally begin after the age of 60 [7, 8]. On average, 5–13% of older adults aged 60–70 are affected by sarcopenia, and its prevalence rises to 11–50% among individuals aged 80 and above. While sarcopenia may lead to frailty, not all sarcopenic patients become frail, with sarcopenia being nearly twice as prevalent as frailty [9]. As individuals age, the incidence of both conditions tends to increase [10, 11]. Multiple adverse outcomes, such as falls [12, 13], disability [14, 15], hospitalization [16, 17], and mortality [18, 19], are associated with sarcopenia and frailty. Despite their significant impact, these conditions are often overlooked or inadequately addressed in clinical settings and remain underrecognized in clinical practice.

The latest International Clinical Practice guidelines strongly recommend the prescription of resistance‐based physical activity as a first‐line nonpharmaceutical treatment among patients with frailty [20] and sarcopenia [21]. Indeed, compelling evidence has suggested that resistance exercise is beneficial for maintaining and improving muscle mass, strength, and physical function [20, 22, 23]. A meta‐analysis examining resistance training studies in older adults found that high‐load resistance training (> 60% 1 repetition maximum [RM]) led to greater muscle strength gains compared to low‐load resistance training (< 50% 1RM). [24] However, many older adults in clinical settings struggle to meet the recommendations of the American College of Sports Medicine and the American Heart Association for engaging in high‐load resistance programs [25, 26, 27]. Moreover, considering the nature of the disease itself as a progressive and generalized musculoskeletal disorder, high‐load resistance training may place excessive mechanical stress on fewer muscle fibres, potentially increasing the risk of injury [28, 29]. An epidemiological study further supported this by revealing that between 1990 and 2007, US emergency departments reported 25 335 weight‐training injuries, where individuals aged 55 and older (18.2%) had a higher incidence of injuries during high‐load resistance training compared to those aged 54 and younger (9.3%) [30]. Therefore, it is necessary to develop a safe, easy‐to‐learn and practical exercise program for older adults with sarcopenia or frailty to maintain muscle function and physical performance.

Mind–body exercise (MBE) is a cost‐effective and convenient form of multicomponent exercise that incorporates breathing control, mental regulation, body movement, and posture control [31, 32]. Research has indicated that MBE may yield comparable benefits to resistance training, with significant improvements in muscle strength and function observed in older adults with frailty (pooled SMD 0.57, 95% CI 0.24 to 0.90 vs. 0.58; 95% CI 0.33 to 0.83) [33]. A recent systematic review and meta‐analysis focusing on the effects of MBE in older adults revealed enhancements in lower body flexibility and lower limb strength in the Yoga group, irrespective of whether they were compared to inactive control groups (controls without physical activity) or active controls (walking or chair‐based aerobics) [34]. These findings can be deduced that MBE is advantageous and should be encouraged as a beneficial exercise regimen for older adults.

To date, there has not been an exhaustive investigation of the impact of MBE on muscle function and physical performance in older adults with sarcopenia and frailty. A previous meta‐analysis examined the effects of Tai Chi, a representative form of MBE, in older adults with sarcopenia and frailty. Subgroup analyses in this study solely compared Tai Chi with passive control and active control groups [35]. Previous research has indicated that factors such as exercise duration, frequency, and type could impact the consistency of outcomes [36, 37]. In clinical settings, the allocation of exercise routines often relies on the clinician's personal preferences and abilities. If so, it is difficult to guarantee whether exercise prescriptions are overestimated or underestimated.

Therefore, the aim of this systematic review was to comprehensively explore the effects of MBE on older adults with sarcopenia and frailty. Additionally, to explore the effect of intervention type, duration, and frequency via subgroup analysis, it aimed to provide a reliable exercise recommendation for the rational selection of MBE and a detailed study design for future studies.

## 2 Methods

### 2.1 Protocol and Registration

This study was registered with International Prospective Register of Systematic Reviews (PROSPERO) (No. CRD42023408907), and the systematic review and meta‐analysis were conducted in accordance with the Preferred Reporting Items for Systematic Reviews and Meta‐Analyses (PRISMA) guidelines.

### 2.2 Search Strategy

The PubMed, Embase, Cochrane Library, Web of Science, PsycInfo, CINAHL, China National Knowledge Infrastructure (CNKI), WanFang Database, and Chinese Scientific Journals Full‐Text Database (VIP) were searched for published RCTs up to February 13, 2024 [33, 38, 39, 40]. No restrictions were applied regarding publication year or language. The utilized search terms are listed in Appendix Table S1.

### 2.3 Eligibility Criteria

The inclusion criteria were as follows: participants aged 60 years or above and diagnosed with either frailty or sarcopenia following specific diagnostic guidelines or criteria; interventions with a minimum of 10 participants and one of the intervention arms involving MBE alone lasting at least 4 weeks; comparisons involving a placebo supplement, including usual care, other forms of exercise alone, or no intervention at all; outcomes that had at least one item, including muscle function (grip strength and muscle mass) and physical performance (gait speed, Chair Rise Test [CRT], Timed Up and Go Test [TUGT], Berg Balance Scale [BBS], 6‐min Walk Test [6mWT], and the Short Physical Performance Battery [SPPB]); and study designs including an RCT. The exclusion criteria included failure to meet the inclusion criteria or involvement in nonhuman research.

### 2.4 Selection Criteria of Studies

RHW and JH conducted a preliminary screening of all the retrieved articles by assessing titles and abstracts, and eliminating duplicate studies. If the topic could not be determined from the title or abstract, the full text was reviewed. Disagreements were resolved through discussion to reach a consensus. If consensus could not be reached, the corresponding author (ZZL) made the final decision on the study eligibility criteria.

### 2.5 Data Extraction

Two authors (RHW and JH) independently completed the data extraction via a standard extraction form designed by DTL and KLW. For each of the included studies, the publication information (e.g. authors, year, and country), participant characteristics (e.g. age, gender, and sample size), study design, intervention (e.g. types, frequency, and duration), and comparison (e.g. passive control and active control), and outcome measurements were extracted.

For the outcome of interest, the mean and standard deviation (mean ± SD) for the outcome of interest were directly extracted from the raw data of the included studies. If the mean and SD were presented in other forms, such as standard errors (SEs), confidence intervals (CIs), or interquartile ranges (IQRs), the RevMan 5.3 calculator was utilized to convert them accordingly. Data at any given time point of interest in each included study were documented. Moreover, the software Engauge Digitizer 10.8 was only used when the outcome was solely presented in a graphical format. In cases where raw data extraction was challenging, the authors of the respective studies were contacted for clarification.

### 2.6 Risk of Bias

The risk of bias in the included studies was evaluated by two independent authors (RHW and JH) via the Cochrane risk of bias tool. Selection bias, performance bias, detection bias, attrition bias, reporting bias, and other bias were included to determine the risk of bias of the included studies. Finally, three categories (‘low risk of bias’, ‘unclear risk of bias’, and ‘high risk of bias’) were used to categorize the risk of bias.

### 2.7 Data Synthesis and Analysis

The meta‐analyses were conducted using Review Manager Version 5.3.0 and STATA Version 16.0 software on Windows. For continuous data, the mean difference (post/preintervention) and standard deviation (SD) of each arm were calculated. The weighted mean difference (WMD) and standardized mean difference (SMD) were utilized to compare the mean values between the intervention and comparison groups based on the similarity of the data rating scales. Heterogeneity was assessed using the *P* value and I^2^ test, with *p* ≥ 0.1 and *I* ^2^ ≤ 50% indicating low heterogeneity. In contrast, when high heterogeneity was detected, a random‐effects model was employed. A funnel plot was used to detect publication bias, and Egger's test was used to confirm the significance of asymmetry. Publication bias was represented by a two‐tailed value of *p* < 0.05. In such instances, the trim and fill methods were used to address publication bias.

Sensitivity analysis was conducted to verify the reliability of the results, and subgroup analyses were performed to investigate factors influencing the impact of MBE on individuals with sarcopenia and frailty, including MBE modes, frequency (1–2 times/week vs. 3–4 times/week vs. ≥ 5 times/week), and duration (4–12 weeks vs. 13–24 weeks vs. > 24 weeks).

## 3 Results

### 3.1 Study Selection

**FIGURE 1:**
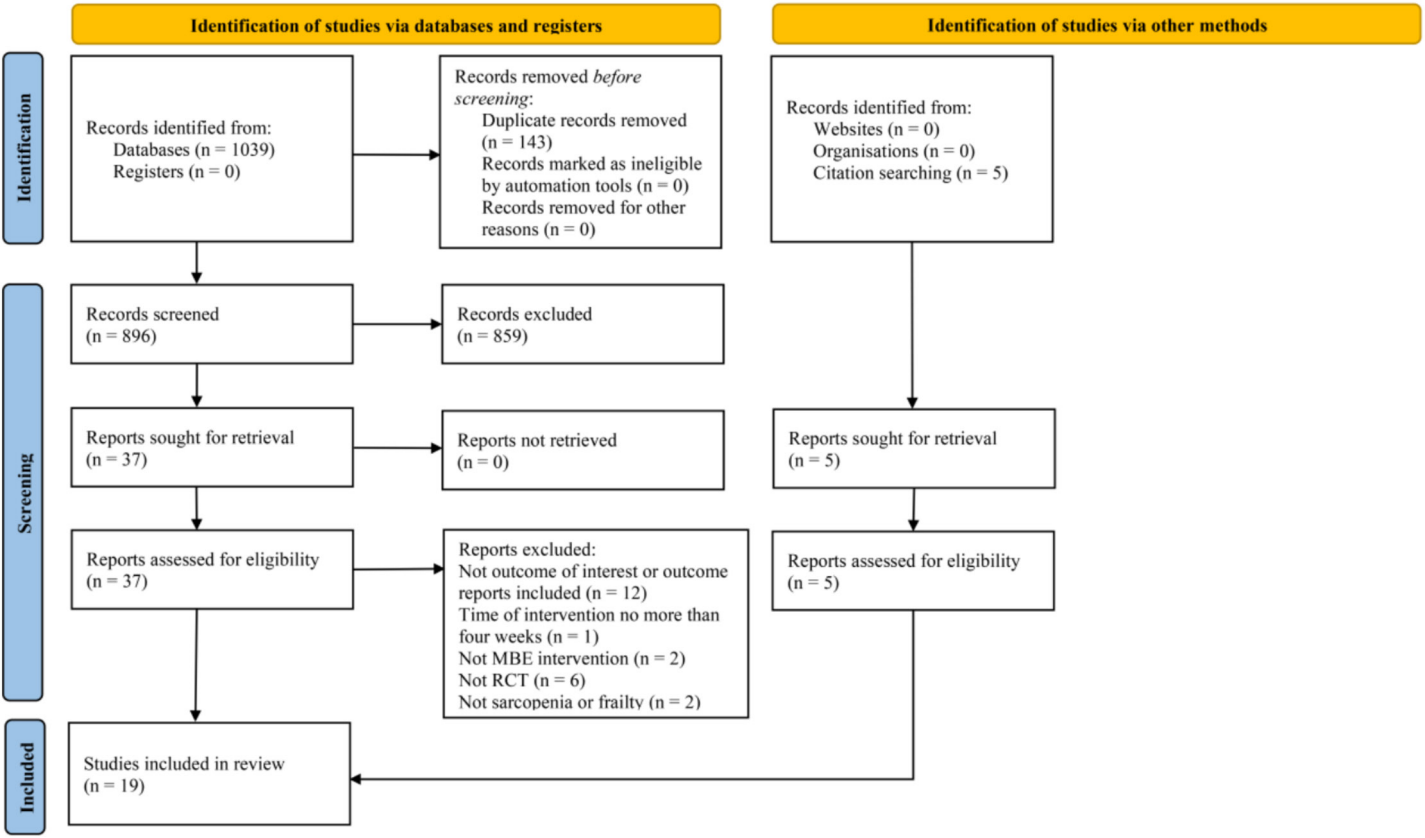
PRISMA flowchart for searching of the included studies.

A total of 1039 potential citations were identified from nine electronic databases and other sources (*n* = 5) using a predefined literature retrieval strategy. After removing 143 duplicate studies, two independent authors (RHW and JH) screened titles and abstracts and discarded obviously irrelevant records. Subsequently, 42 studies underwent full‐text review for potential eligibility, with 19 articles excluded for the following reasons: lack of relevant outcomes or outcome reporting (*n* = 12), interventions lasting less than 4 weeks (*n* = 1), interventions not related to MBE (*n* = 2), non‐RCT design (*n* = 6), and not focusing on sarcopenia or frailty (n = 2). Ultimately, 19 studies met the inclusion criteria for qualitative and quantitative analysis. The detailed screening flow is presented in Figure 1.

### 3.2 Study Characteristics

The characteristics of each eligible study are summarized in Table 1. A total of 19 studies with 1838 participants were included, with participant ages ranging from 60 to 95 years. The studies were conducted in various locations, including Georgia (*n* = 2) [41, 42], Mainland China (*n* = 12) [43, 44, 45, 46, 47, 48, 49, 50, 51, 52, 53, 54], Hong Kong (*n* = 1) [55], France (*n* = 1) [56], Australia (*n* = 1) [57], UK (*n* = 1) [58], and Poland (*n* = 1) [59]. The forms of MBE in the experimental group were diverse, such as Tai Chi [41, 42, 44, 49, 51, 52, 56, 57, 59, 60], Yijinjing [43, 46, 47, 53, 54], Baduanjin [45, 50], and Yoga [57], with 15 studies [41, 42, 43, 44, 46, 47, 49, 51, 52, 53, 54, 55, 56, 57, 58] mentioning expert instruction in the MBE program. Tai Chi (*n* = 12) was the predominant form of MBE [43, 44, 45, 46, 47, 48, 49, 50, 51, 52, 53, 54], encompassing various schools, with the majority of participants practising Yang‐style MBE (*n* = 9) [41, 42, 44, 49, 51, 52, 56, 58, 59] and only one study not specifying the Tai Chi style [57].

**TABLE 1:** The characteristics of included studies.

Studies	Country/origin	Participants (age: years)	Gender (M/F)	Sample size (pre‐/post‐)	Study design	Intervention (types/frequency/duration of MBE); expert	Control	Outcome measures
Jin et al., 2008	China	Older adults with sarcopenia IC: 68.22 ± 4.09 CC: 65.09 ± 3.95	21/50	IC: 36/36 CC: 35/35	RAN‐PA	Yijinjing 60 min/session, 3 sessions/week, total 8 weeks Yijinjing expert	Usual care	1. Physical performance: 6mWT, 15 s Chair rise test 2. Grip strength
Wang et al., 2012	China	Older adults with sarcopenia IC: 66.79 ± 4.76 CC: 65.59 ± 3.59	22/53	IC: 38/38 CC: 37/37	RAN‐PA	Yijinjing 60 min/session, 3 sessions/week, total 12 weeks Yijinjing expert	Usual care	1. Grip strength 2. Physical performance: 15 s CRT
Zhu et al., 2019	China	Older adults with sarcopenia IC: 65.60 ± 11.36 CC: 66.32 ± 10.80	32/31	IC: 32/32 CC: 31/31	RAN‐PA	Yijinjing 40 min/session, 7 session/week, total 12 weeks Yijinjing expert	Usual care	1. Grip strength 2. Physical performance: 15 s CRT
Zhu2 et al., 2019	China	Older adults with sarcopenia IC: 88.8 ± 3.7 CC1: 89.5 ± 4.4 CC2: 87.5 ± 3.0	79/0	IC: 30/24 CCI: 30/28 CC2: 30/27	RAN‐PA	Tai Chi (Yang‐ style): 40 min/session, 5 sessions/week, total 8 weeks Tai Chi expert	CC1: whole‐body vibration CC2: health education	1.Muscle mass, grip strength 2. Physical performance: balance, gait speed, TUGT, CRT
Zhou et al., 2020	China	Older adults with sarcopenia IC: 72.67 ± 9.56 CC: 73.25 ± 8.54	17/23	IC: 20/20 CC: 20/20	RAN‐PA	Baduanjin 40 min/session, 5 sessions/week, total 8 weeks Expert: no mention	Usual care	1. Grip strength 2. Physical performance: 30 s CRT, TUGT, BBS
Fang et al., 2020	China	Older adults with sarcopenia IC: 82.8 ± 8.5 CC: 76.3 ± 9.9	12/24	IC: 18/15 CC: 18/14	RAN‐PA	Yijinjing 25–30 min/session, 3 sessions/week, total 6 months Yijinjing expert	Health education	1. Physical performance: TUGT
Morawin et al., 2021	Poland	Older adults with sarcopenia 70.5 ± 5.8	No mention	IC: 40/23 CC: 40/21	RAN‐PA	Tai Chi (Yang‐ style) 40 min/session, 2 sessions/week, total 10 months Expert: no mention	Health education	1. Muscle mass, grip strength 2. Physical performance: gait speed, 6mWT
Peng et al., 2022	China	Older adults with sarcopenia IC: 72.12 ± 6.47 CC: 71.85 ± 5.73	33/47	IC: 40/39 CC: 40/38	RAN‐PA	Yijinjing 45 min/session, 5 sessions/week, total 8 weeks Yijinjing expert	Exercise therapy	1. Physical performance: gait speed, BBS, 6mWT
Li et al., 2022	China	Older adults with sarcopenia IC: 80.57 ± 8.93 CC: 77.89 ± 10.38	21/49	IC: 35/35 CC: 35/35	RAN‐PA‐SB	Ditangquan 60 min/session, 3 sessions/weeks, total 24 weeks Expert: no mention	Conventional exercises	1. Physical performance: TUGT
Wolf et al., 1996	Georgia	Older adults with frailty IC: 76.9 ± 4.8 CC1: 76.3 ± 5.1 CC2: 75.4 ± 4.1	39/161	IC: 72/58 CC1: 64/50 CC2: 64/54	RAN‐PA‐SB	Tai Chi (Yang‐style) 30 min/session, 7 sessions/week, total 15 weeks Tai Chi expert	CC1: balance training CC2: health education	1. Grip strength 2. Physical performance: gait speed
Wolf et al., 2006	Georgia	Older adults with frailty IC: 81.0 ± 6.4 CC: 80.8 ± 5.9	20/291	IC: 158/107 CC: 153/106	RAN‐PA‐SB	Tai Chi (Yang‐style) 60 min/session, 2 sessions/week, total 12 months Tai Chi expert	Usual care	1.Physical performance: gait speed, three chair stand
Dechamps et al., 2009	France	Older adults with frailty IC: 80.8 ± 8.7 CC: 80.6 ± 9.2	22/30	IC: 26/15 CC: 26/21	RAN‐PA‐SB	Tai Chi (Yang‐style) 30 min/session, 4 sessions/week, total 24 weeks Tai Chi expert	Cognition‐action exercise	1. Physical performance: TUGT, balance
Tsang et al., 2013	Hong Kong, China	Older adults with frailty IC: 83.33 ± 6.30 CC: 84.85 ± 6.03	19/87	IC: 69/61 CC: 65/55	RAN‐PA‐SB	Qigong 60 min/session, 2 sessions/week, total 12 weeks Qigong expert	Usual care	1. Grip strength 2. Physical performance: TUGT
Saravanakumar et al.,2014	Australia	Older adults with frailty IC1: 81.1 ± 8.0 IC2: 84.9 ± 6.7 CC: 85.4 ± 9.1	9/24	IC1: 11/9 IC2: 11/8 CC: 11/9	RAN‐PA‐SB	IC1: Tai Chi (No mention) 30 min/session, 2 sessions/week, total 14 weeks IC2: Yoga 30 min/session, 2 sessions/week, total 14 weeks Tai Chi and Yoga experts	Usual care	1.Physical performance: BBS
Kasim et al., 2020	UK	Older adults with frailty IC: 71 ± 3.1 CC: 70 ± 3.6	5/16	IC: 11/11 CC: 10/10	RAN‐PA	Tai Chi (Yang‐ style): 60 min/session, 3 sessions/week, total 12 weeks Tai Chi expert	Zumba Gold®	1.Physcal performance: TUGT, 30‐s CRT
Meng et al., 2022	China	Older adults with frailty IC1: 75.60 ± 1.92 IC2: 76.31 ± 2.07 CC: 76.59 ± 2.58	66/84	IC1:50/50 IC2:50/50 CC:50/50	RAN‐PA‐DB	IC1: Tai Chi (Yang‐style) IC2: Tai Chi+ strength and endurance training 60 min/session, 3 sessions/week, total 24 weeks Tai Chi expert	Strength and endurance training	1. Grip strength 2. Physical performance: gait speed, TUGT, 6mWT
Wang et al., 2022	China	Older adults with frailty IC1: 71.84 ± 3.77 IC2: 70.65 ± 3.73 CC: 70.74 ± 3.52	80/91	IC1: 57/57 IC2: 57/57 CC: 57/57	RAN‐PA‐DB	IC1: Baduanjin IC2: Baduanjin + strength and endurance training 60 min/session, 3 sessions/week, total 24 weeks Expert:No mention	Strength and endurance training	1. Grip strength 2. Physical performance: gait speed, TUGT, 6mWT
Ge et al., 2021	China	Older adults with Per‐frailty IC: 70.16 ± 5.40 CC: 72.91 ± 6.61	28/37	IC: 32/32 CC: 33/33	RAN‐PA‐SB	Tai Chi (Yang‐style): 60 min/time, 3 sessions/week, total 8 weeks Tai Chi expert	Usual care	1.Physical performance: 30‐s CRT, gait speed
Zhang et al., 2022	China	Older adults with Per‐frailty IC1: 71.3 ± 5.0 IC2: 71.7 ± 3.9 CC: 70.8 ± 4.2	37/51	IC1: 31/31 IC2: 31/30 CC: 31/30	RAN‐PA‐SB	IC1: Tai Chi (Yang‐style) + Mindfulness IC2: Tai Chi 60 min/session, 2 sessions/week, total 12 weeks Tai Chi expert	Mindfulness	1. Physical performance: SPPB, TUGT, 30‐s CRT

*Note:* The numeral ‘2’ in Zhu2 et al., 2019 is appended to differentiate between two studies by authors sharing the surname Zhu and published in the same year (2019).

Abbreviations: 6mWT, 6‐min Walk Test; BBS, Berg Balance Scale; CC, control cohort; CRT, Chair Rise Test; DB, double‐blind; IC, intervention cohort; MBE, mind–body exercise; PA, parallel; RAN, randomized; SB, single‐blind; SPPB, Short Physical Performance Battery; TUGT, Timed‐Up‐and‐Go Test.

Among the included studies, 12 studies compared MBE with passive controls (usual care or health education) [41, 42, 43, 44, 45, 46, 51, 53, 54, 55, 57, 59], whereas 9 studies compared MBE with active controls (whole‐body vibration, cognition‐action exercise, and balance training, among other procedures) [41, 44, 47, 48, 49, 50, 52, 56, 58]. The frequency of MBE sessions ranged from 2 to 7 times per week, thus lasting between 25 and 90 min per session, with intervention durations varying from 8 to 40 weeks. The key measured outcomes included grip strength and muscle mass for muscle function, as well as the TUGT, CRT, BBS, 6mWT, and gait speed for physical function.

### 3.3 Risk of Bias

**FIGURE 2:**
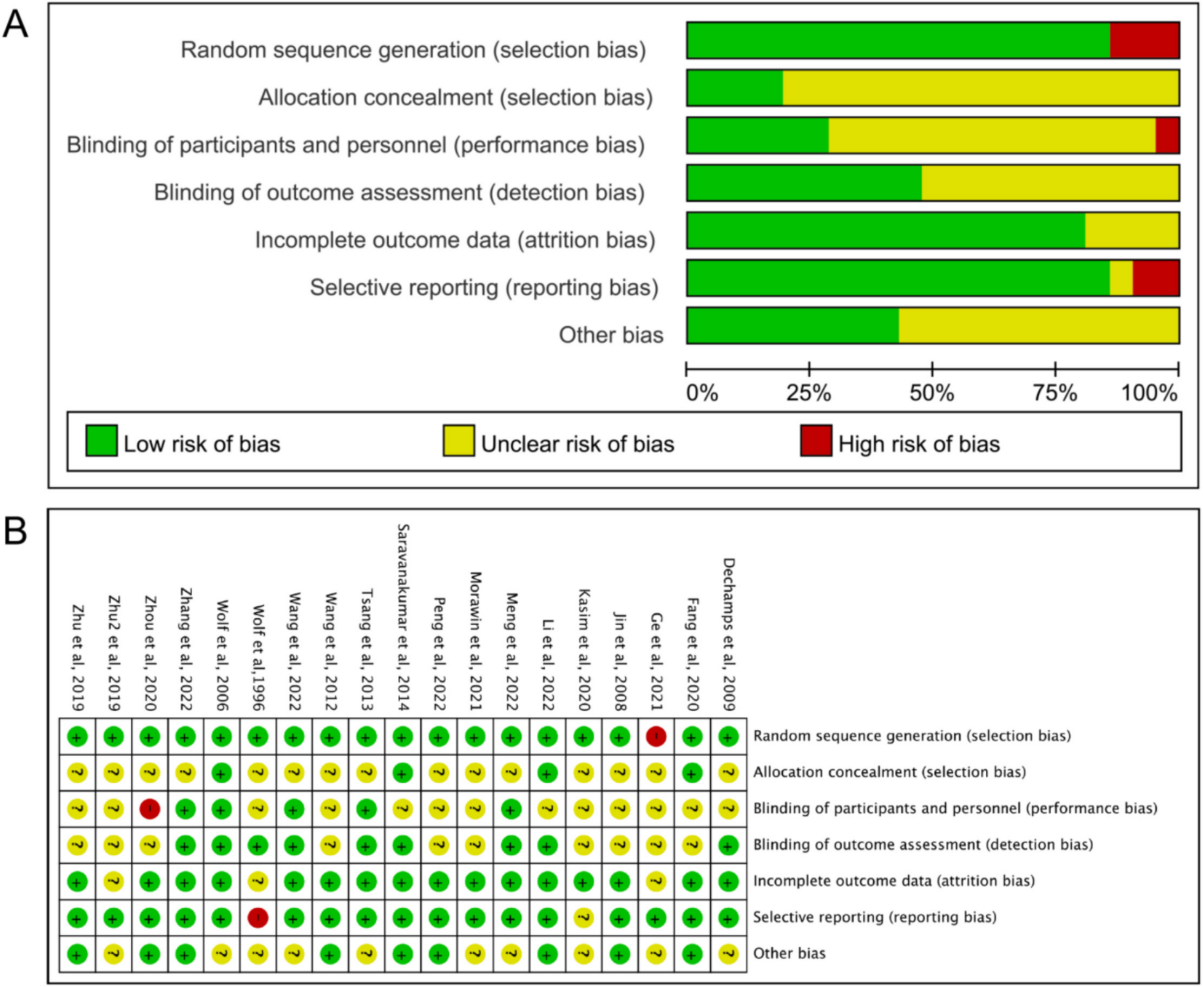
Risk of bias graph and summary of each included study. The numeral ‘2’ in Zhu2 et al., 2019 is appended to differentiate between two studies by authors sharing the surname Zhu and published in the same year (2019).

The risk of bias in the included studies was assessed using the Cochrane risk of bias tool by two independent authors (RHW and JH) following the guidelines outlined in Section 8 of the Cochrane Handbook for Systematic Reviews of Interventions (Figure 2A,B) [61]. Out of the 19 studies, only one study did not report the randomization allocation methods such as random number tables and computer generation [51], and four studies explicitly mentioned allocation concealment by keeping allocation numbers in sealed containers [42, 46, 48, 57]. Regarding potential performance bias, one study showed a high risk [45], whereas 10 studies had an unclear risk due to difficulties in blinding participants, personnel, and staff to exercise interventions [43, 44, 45, 46, 47, 51, 53, 54, 58, 59]. In contrast, nine studies blinded the outcome assessors [41, 42, 48, 49, 50, 52, 55, 56, 57], resulting in a low risk of detection bias. Attrition bias was low in 16 studies [42, 43, 45, 46, 47, 48, 49, 50, 52, 53, 54, 55, 56, 57, 59, 60], including complete research data and detailed descriptions of participant dropouts or follow‐up failures. The handling of incomplete data in the remaining studies was deemed to be appropriate. Selective reporting bias in two studies was high or unclear [58, 62], as 17 trials analysed and reported outcomes in conjunction with their methods or publicly available protocols. Finally, the risk of other bias in nine studies was low [43, 45, 46, 47, 48, 52, 53, 54, 57].

### 3.4 Effectiveness of MBE on Outcomes

#### 3.4.1 MBE vs. Passive Control

The impacts of MBE compared to passive control on various aspects of muscle function, such as grip strength and muscle mass, as well as physical performance measures such as the CRT, TUGT, gait speed, 6mWT, and BBS, were assessed.

**FIGURE 3:**
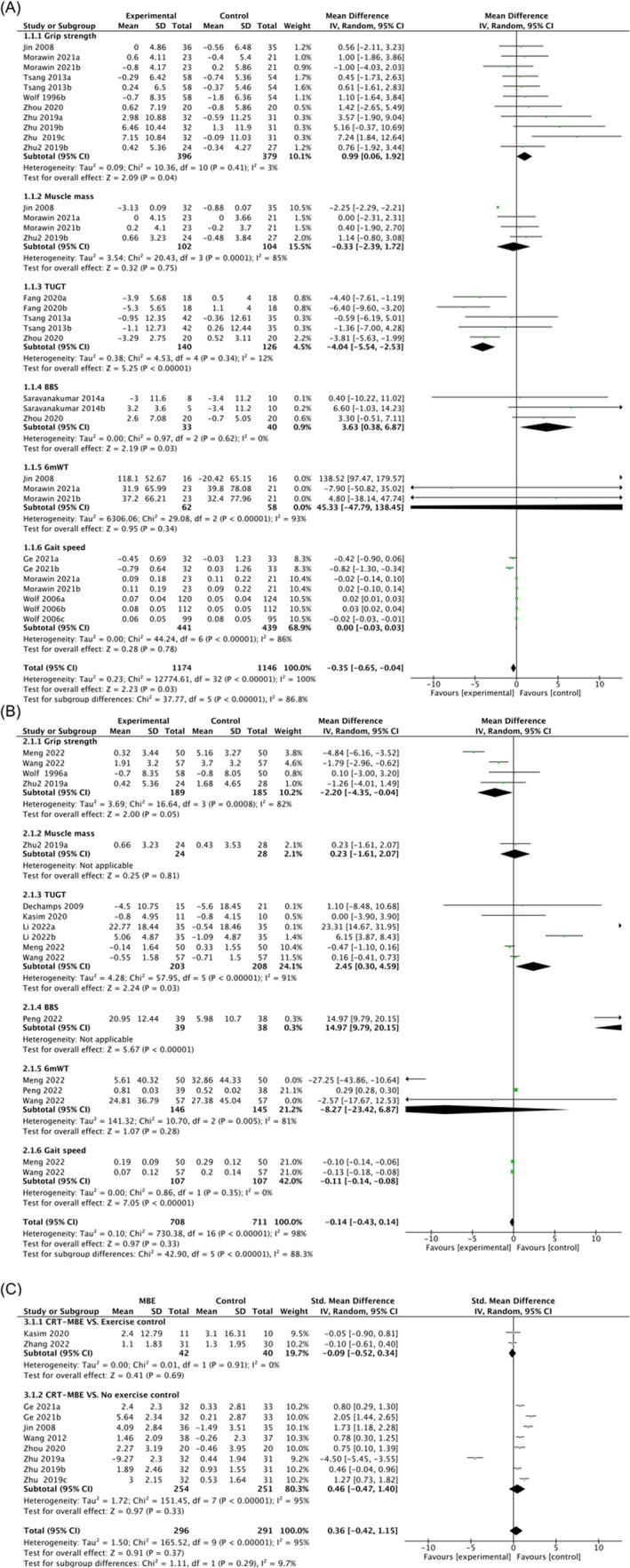
Forest plots for comparing MBE with different control groups on various indicators. (A) MBE vs. passive control; (B) MBE vs. active control; (C) MBE vs. passive control/active control on BBS. The numeral ‘2’ in Zhu2 et al., 2019 is appended to differentiate between two studies by authors sharing the surname Zhu and published in the same year (2019). The letters a, b, and c represent the first, second, and third intervention time points, respectively. MBE, mind–body exercise; TUGT, Timed‐Up‐and‐Go test; BBS, Berg Balance Scale; CRT, Chair Rise Test; 6mWT, 6‐min Walk Test.

For muscle function (Figure 3A), in a review of seven studies encompassing 11 comparisons and a total of 775 participants, the effects of MBE compared with passive control were examined in relation to grip strength, with interventions including Yijinjing, Tai Chi, and Baduanjin. The meta‐analysis demonstrated a significant improvement in grip strength with MBE intervention (WMD = 0.99; 95% CI = 0.06, 1.92; *I* ^2^ = 3%, *p* = 0.04). Additionally, three studies involving 206 patients with sarcopenia and frailty examined the impact of MBE interventions (specifically, Yijinjing and Tai Chi) on muscle mass compared to passive control. Despite high heterogeneity (*I* ^2^ = 85%), no significant improvement was observed (WMD = −0.33; 95% CI = −2.39, 1.72; *p* = 0.75). A sensitivity analysis was conducted by systematically removing individual studies, thus demonstrating that the heterogeneity was primarily due to Jin et al.’s study (2008). Upon exclusion of this study, the heterogeneity decreased significantly (*I* ^2^ = 0%), whereas the overall results remained stable (*p* = 0.36), thus indicating the robustness of the statistical findings.

For physical performance (Figure 3A,C), three studies involving 266 patients with sarcopenia and frailty assessed the effect of MBE interventions by using Yijinjing, Qigong, and Baduanjin on TUGT results compared to the passive control. The meta‐analysis demonstrated a statistically significant positive effect of MBE on the TUGT results (WMD = −4.04; 95% CI = −5.54, −2.53; *I* ^2^ = 12%, *p* < 0.001). Furthermore, an evident discrepancy was found in the BBS score (WMD = 3.63; 95% CI = 0.38, 6.87; *I* ^2^ = 0%, *p* = 0.03). The results of CRT were assessed in five studies with 505 participants, and no positive effect was observed for this indicator, as well as with high heterogeneity (SMD = 0.46; 95% CI = −0.47, 1.40; *I* ^2^ = 95%, *p* = 0.33) (Figure 3C). Sensitivity analysis demonstrated a minimal change in effect size upon exclusion of any individual study, with heterogeneity persisting at a high level. A similar result was observed for the 6mWT (WMD = 45.33; 95% CI = −47.79, 138.45; *I* ^2^ = 93%, *p* = 0.34). In terms of gait speed, three studies encompassing seven comparisons comparing MBE by using Tai Chi with passive control indicated no significant improvement and high heterogeneity (*I* ^2^ = 86%, *p* = 0.78).

The GRAGE recommendations for muscle function and physical performance in older adults with sarcopenia and frailty in the MBE group compared to those in the passive control group are detailed in Table S2 of supplementary Appendix 1.

#### 3.4.2 MBE vs. Active Control

Nine of the included studies compared MBE to active control, such as balance training, cognition‐action exercise, and whole‐body vibration, by using interventions such as Tai Chi, Yijinjing, Baduanjin, and Ditangquan (Figure 3B,C).

A random effects model was utilized for the high variability outcomes, thus yielding a WMD of −2.20 (95% CI = −4.35, −0.04; *p* = 0.05) for grip strength, 2.45 (95% CI = 0.30, 4.59; *p* = 0.03) for the TUGT results, an SMD of −0.09 (95% CI = −0.52, 0.34; *p* = 0.69) for CRT, and a WMD of −0.11 (95% CI = −0.14, −0.08; *I* ^2^ = 0%, *p* < 0.001) for gait speed.

Additionally, only one study reported a positive effect of MBE on BBS scores compared to the active control group (WMD = 14.97; 95% CI = 9.79, 20.15; *p* < 0.001). One study evaluated the effect of MBE versus active control on muscle mass, showing no significant improvement (WMD = 0.23; 95% CI = −1.61, 2.07; *p* = 0.81). Two studies involving three results indicated no significant improvement in the 6mWT (WMD = −8.27; 95% CI = −23.42, 6.87; *I* ^2^ = 81%, *p* = 0.28). Sensitivity analysis demonstrated decreased heterogeneity after excluding the study by Meng et al. (2022) (*p* < 0.001).

### 3.5 Subgroup Analysis

After conducting further screening, a subgroup analysis was performed to examine the effects of exercise prescriptions on muscle function and physical performance. This analysis specifically focused on grip strength, gait speed, TUGT, and CRT. The results are shown in Table S3 of Supplementary Appendix 1.

For muscle function, Yijinjing was more effective for improving grip strength in patients with sarcopenia and frailty when compared with the passive control (WMD = 1.27; 95% CI = 0.04, 2.51; *I* ^2^ = 41%, *p* = 0.04) (Figure S1). A high frequency of exercise ≥ 5 times a week had a significant effect on muscle function according to grip strength measurements, whereas no positive effect size was found for low‐frequency exercise (1–2 times/week) (WMD = 0.31; 95% CI = −0.91, 1.53; *I* ^2^ = 0%, *p* = 0.62) or moderate‐frequency exercise (3–4 times/week) (WMD = 0.56; 95% CI = −2.11, 3.23; *p* = 0.68) (Figure S2). Both short (4–12 weeks) (WMD = 1.35; 95% CI = 0.10, 2.59; *I* ^2^ = 18%, *p* = 0.03) and medium (13–24 weeks) (WMD = 41.61; 95% CI = 5.94, 77.28; *p* = 0.02) durations of MBE significantly affected grip strength (Figure S3).

For physical function, Baduanjin was recommended to improve lower limb support strength and performance via the use of the CRT (WMD = 0.75; 95% CI = 0.10, 1.39; *p* = 0.02) and TUGT (WMD = −3.81; 95% CI = −5.63, −1.99; *p* < 0.001). Moreover, Tai Chi (WMD = 1.41; 95% CI = 0.18, 2.63; *p* = 0.02) and Yijinjing (WMD = −4.32; 95% CI = −6.29, −2.35; *I* ^2^ = 32%, *p* < 0.001) have also been shown to improve these two indicators, respectively (Figures S4 and S6). Similarly, a moderate frequency (3–4 times/week) (WMD = 1.32; 95% CI = 0.70, 1.94; *p* < 0.001) of MBE significantly improved physical function by reducing the duration of CRT (Figure S5) and enhancing gait speed (WMD = −0.62; 95% CI = −1.01, −0.23; *I* ^2^ = 24%, *p* = 0.002) (Figure S8). However, this frequency of MBE showed a high degree of heterogeneity in CRT (*I* ^2^ = 82%). After removing the studies in sequence, the heterogeneity remained almost unchanged. Notably, the combined results did not change, and the statistical results were stable. No positive effects were detected at a high frequency (≥ 5 times a week) (Figure S5). After excluding the study by Zhou et al. (2019a), the heterogeneity decreased to a low level in the sensitivity analysis (*p* = 0.001). In addition, all of the studies that included CRT included short‐term interventions (4–12 weeks). For gait speed, the low frequency of MBE also showed high heterogeneity (Figure S8). After removing the studies by Wolf et al. (2006c), the heterogeneity decreased (*I* ^2^ = 0%, *p* < 0.001). Similarly, both short (4–12 weeks) and medium (13–24 weeks) durations of exercise produced significant improvements in gait speed (WMD = −0.62; 95% CI = −1.01, −0.23; *I* ^2^ = 24%, *p* = 0.002) (Figure S9) and TUGT (WMD = −6.40; 95% CI = −9.60, −3.20; *p* < 0.001) (Figure S7). Among them, no significant results were found for studies lasting longer than 24 weeks for gait speed, and there was high heterogeneity. However, the heterogeneity could be zeroed out when excluding the Wolf et al.’s study (2006c). All of the forest plots for the above‐mentioned subgroup analysis (Figures S1–S9) are provided in Supplementary Appendix 2.

### 3.6 Publication Bias

**FIGURE 4:**
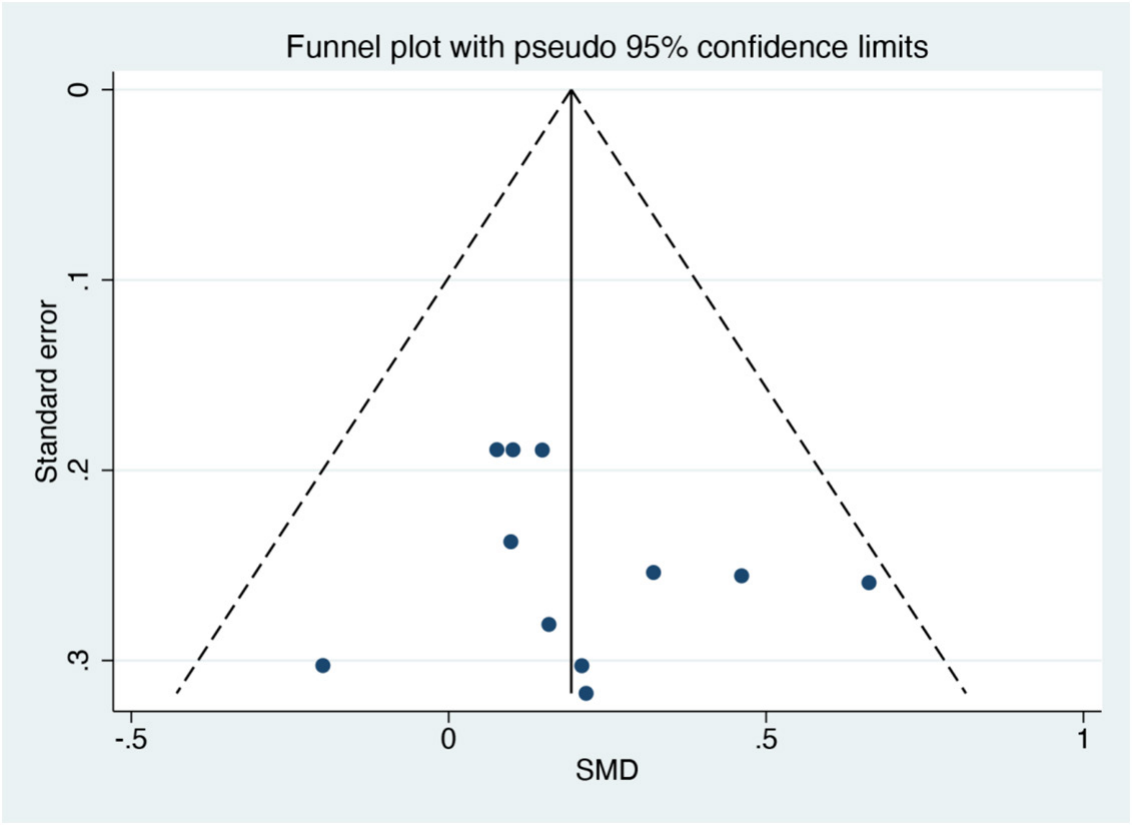
Publication bias funnel plots of standard errors and effect sizes of the included studies. SMD, standard mean difference.

Funnel plots were generated to determine the risk of publication bias for grip strength, which was reported in more than 10 studies (Figure 4). Noticeable symmetries were observed in these funnel plots, indicating no obvious publication biases regarding MBE intervention for grip strength among patients with sarcopenia and frailty. Furthermore, the Egger's test results showed good grip strength (*p* = 0.557), thus suggesting that our results were robust.

## 4 Discussion

This study is one of the first to investigate the impact of MBE on muscle function and physical performance in older adults with sarcopenia and frailty. Additionally, this study aimed to identify the key factors influencing exercise prescriptions that have maximize improvements in muscle function and physical performance, which is crucial for developing reliable exercise recommendations and detailed study protocols for future investigations. The meta‐analysis demonstrated several important findings. First, MBE interventions demonstrated greater effectiveness than passive control in enhancing muscle function and physical performance, particularly in terms of grip strength, TUGT, and BBS scores. Second, subgroup analysis focusing on muscle function indicated that Yijinjing was particularly beneficial for improving grip strength in individuals with sarcopenia and frailty when performed more than 5 times per week for short (4–12 weeks) or medium (13–24 weeks) durations. Moreover, for enhancing physical performance outcomes, such as gait speed, TUGT, and CRT, MBE interventions were effective even with low (1–2 times/week) or moderate (3–4 times/week) training frequencies when intervention duration was comparable. However, the study did not find evidence indicating that MBE significantly increases muscle mass.

Multiple guidelines for the diagnosis of sarcopenia and frailty consistently emphasize that muscle strength, muscle mass, and physical performance are key factors affected by these conditions [3, 20, 21, 63]. This is the reason why this study focused on exploring the effects of MBE on muscle function (muscle strength and muscle mass) and physical performance. Among them, grip strength, which is a simple and effective measure of upper‐limb strength, is widely used to assess muscle strength [1]. Evidence from 12 British studies underscores the importance of grip strength and muscle mass in the context of sarcopenia and frailty phenotypes [64]. The pooled analysis results of this study revealed a significant improvement in grip strength following MBE interventions, but no significant improvement in muscle mass was observed. This finding aligns with Huang et al.’s study regarding muscle mass results but differs in terms of grip strength [35]. A previous review showed that engaging in resistance training more than three times a week for a minimum of 12 weeks could enhance grip strength in patients with sarcopenia [65]. This study indicated that engaging in MBE more than 5 times a week for a short or medium duration (4–24 weeks) could improve grip strength. This finding implies that MBE may provide comparable benefits to resistance exercise. Furthermore, training frequency may be a crucial determinant in enhancing grip strength, and regular MBE, even in short or moderate training cycles, may lead to positive results.

Further subgroup analyses from the forest plot on the effects of different types of MBE on grip strength indicated that only Yijinjing showed improvement in muscle strength, whereas Tai Chi and Baduanjin did not demonstrate the same effect (Figure S1). This discrepancy could be attributed to the specific characteristics of Tai Chi, which primarily focuses on slow and continuous weight‐shifting exercises for the lower limbs, with minimal emphasis on hand strength exercises [66, 67]. Previous studies have further underscored the positive impact of Tai Chi on lower limb muscles, particularly the knee extensor groups like the quadriceps [68]. The pooled results from a systematic review and meta‐analysis of 3 out of 14 RCTs indicated that Baduanjin training significantly improved grip strength [69]. Variations in study population concerning health status, sex, and age range may account for the discrepancies in findings. Notably, all three studies focused on middle‐aged and elderly participants in good health [70, 71, 72], with two exclusively involving male participants [71, 72]. In contrast, the study by Zhou et al. [45] concentrated on individuals over 60 years of age with sarcopenia, who may have had various comorbidities or chronic conditions that could influence the effectiveness of Baduanjin training. These inconsistencies across studies may partially reflect the confounding influence of population characteristics on grip strength outcomes.

Physical performance, which is a key hallmark of sarcopenia and frailty [73], was regarded as being the primary driver of the associations of sarcopenia and frailty with adverse health outcomes [74]. The assessments included tests such as the TUGT, CRT, gait speed, 6mWT, and BBS in this study. Consistent with previous research [75], a positive impact was observed on TUGT. A systematic review and meta‐analysis examining the effects of various exercise regimens on muscle strength and physical performance in older adults with sarcopenia revealed significant enhancements in TUGT across all exercise modes [76]. This finding suggests that TUGT may serve as a more sensitive measure of exercise‐induced improvements. However, the outcomes for gait speed and CRT were inconsistent in this study and also displayed a relatively high degree of heterogeneity. These metrics are commonly used to evaluate physical performance, with improvements potentially leading to enhanced independence and reduced risk of adverse events [74, 77]. Subgroup analyses further suggested that variations in gait speed and CRT results might be linked to differences in intervention duration and/or frequency. Specifically, a moderate‐frequency intervention over a short period in older adults with sarcopenia and frailty yielded greater improvements in gait speed and CRT. This implies that a moderate‐frequency intervention could be an efficacious approach to enhancing mobility in this population within a short timeframe. Nevertheless, it is crucial to acknowledge the GRADE evidence level for gait speed and CRT was relatively low, necessitating the cautious interpretation of these findings.

When the study focused on the effects of MBE on muscle function and physical performance in older adults with sarcopenia and frailty compared to active exercise training, the results showed that MBE could enhance physical performance, including TUGT, BBS, and gait speed, and have a trend of improvement in grip strength. These findings suggest that MBE can enhance muscle function and overall physical performance to some extent in this population, regardless of whether they are compared with passive or active exercise training. Therefore, MBE should be considered a viable exercise option for this population. To further validate the effectiveness of MBE, studies are needed to compare it with well‐designed active exercise training programs that match the exercise volume. An analysis of five studies [44, 47, 52, 56, 60] focusing on MBE versus active exercise training that match the exercise volume revealed significant improvements in BBS and 6mWT within the same study. However, the limited number of eligible studies for each indicator constrains the ability to draw definitive conclusions. Future studies should include more comparable research to strengthen the evidence supporting the efficacy of MBE.

Numerous age‐related changes associated with sarcopenia and frailty primarily affect skeletal muscle [65]. These changes encompass alterations in body fat distribution, ectopic fat accumulation, chronic low‐grade inflammation, modifications in muscle structure, declines in muscle mass and function, and mitochondrial dysfunction [78, 79, 80, 81]. These alterations have significant implications for muscle metabolism and insulin sensitivity [82]. Several studies have suggested that these changes may be influenced or worsened by physical inactivity rather than just by ageing itself. Exercise can potentially enhance and regulate multiple systems simultaneously by triggering the production and release of numerous myokines, which play a crucial role in maintaining overall metabolic balance [81, 83, 84]. Hence, the enhancement of muscle function and physical performance through exercise may be mediated by myokine modulation.

This study is a significant contribution because it offers the first comprehensive and up‐to‐date review and meta‐analysis of RCTs on MBE interventions that specifically target muscle function and physical performance in adults aged 60 years and older with sarcopenia and frailty. The study utilized a rigorous search strategy to gather relevant data. However, this meta‐analysis had several limitations. First, the overall quality of some of the included studies was deemed to be generally poor, which is a common issue in reviews of this nature [35, 75, 85]. Second, although the study encompasses articles from six countries, including Georgia, China, France, England, Australia, and Poland, to showcase the global impact of MBE, it is important to note that the research sample in this study exhibits a high level of homogeneity, with the majority of studies originating from China. This may be attributed to increasing popularity of MBE in China. Asia, where Tai Chi [86], Baduanjin [87], Qigong [87], and Yoga [88] originated. This cultural familiarity may lead to a deeper understanding of the nuances of these practices. Furthermore, a qualitative study involving 15 academic physicians from non‐Asian countries indicated that nearly three‐quarters of the physicians considered Tai Chi, a representative form of MBE, to be a potential therapeutic strategy. However, about one‐third of the physicians were never willing to recommend it [89]. Obviously, cultural differences potentially impact the willingness to recommend such practices. While these may limit result generalization, the results remain reliable, unbiased, and still offer valuable insights for future exercise prescription recommendations related to MBE. Third, although subgroup analysis was conducted to identify potential sources of heterogeneity, the number of available studies was limited, thus making it challenging to assess the effects of various prescriptions and potentially impacting the precision of the pooled results. Therefore, further high‐quality clinical trials are necessary to validate the effectiveness of MBE and determine specific exercise parameters.

## 5 Conclusions

This study demonstrated that MBE can enhance muscle function and physical performance to some extent in older adults with sarcopenia and frailty, whether compared with passive or active exercise training. However, no positive effects on muscle mass were observed. Compared to passive control, MBE interventions significantly improved grip strength, TUGT, and BBS scores in this population. This study highlighted that MBE interventions lasting between 4 and 24 weeks, with a frequency of more than 5 times per week, were associated with greater improvements in grip strength. Moreover, a similar intervention period was effective with fewer sessions to enhance physical function, such as gait speed, TUGT, and CRT. This review contributes valuable scientific evidence on the effects of MBE on muscle function and physical performance in patients with sarcopenia and frailty, further high‐quality RCTs (based on the Cochrane tool's assessment of low risk of bias) are necessary to confirm or challenge these results.

## Ethical Statement

The manuscript does not contain clinical studies or patient data.

## Conflicts of Interest

The authors declare no conflicts of interest.

## Supporting information


**Appendix Table S1** Search strategy.
Appendix Table S2 GRADE assessments. Reference: Romina Brignardello‐Petersena, Reem A. Mustafa, Reed A.C. Siemieniuk, M. Hassan Murad, Thomas Agoritsasa, Ariel Izcovich, Holger J. Sch€unemann, Gordon H. Guyatta, for the GRADE Working Group. GRADE approach to rate the certainty from a network meta‐analysis: addressing incoherence. J Clin Epidemiol. 2019 Apr; 108:77–85. doi: 10.1016/j.jclinepi.2018.11.025. Epub 2018 Dec 5.
Appendix Table S3 Results of subgroup analysis based on exercise prescriptions. CRT, Chair Rise Test; TUGT, Timed‐Up‐and‐Go Test.
Figure S1 Forest plot for different exercise types on grip strength.
Figure S2 Forest plot for different exercise frequency on grip strength.
Figure S3 Forest plot for different exercise duration on grip strength.
Figure S4 Forest plot for different exercise types on CRT..CRT, Chair Rise Test.
Figure S5 Forest plot for different exercise frequency on CRT..CRT, Chair Rise Test.
Figure S6 Forest plot for different exercise types on TUGT.TUGT, Timed Up and Go Test.
Figure S7 Forest plot for different exercise duration on TUGT..TUGT, Timed Up and Go Test.
Figure S8 Forest plot for different exercise frequency on gait speed.
Figure S9 Forest plot for different exercise duration on gait speed.
