# Correction to “Effectiveness of Mind–Body Exercise in Older Adults With Sarcopenia and Frailty: A Systematic Review and Meta‐Analysis”

**DOI:** 10.1002/jcsm.13875

**Published:** 2025-07-09

Wan, R. , Huang, J. , Wang, K. , Long, D. , Tao, A. , Huang, J. and Liu, Z. (2025), Effectiveness of Mind–Body Exercise in Older Adults With Sarcopenia and Frailty: A Systematic Review and Meta‐Analysis. Journal of Cachexia, Sarcopenia and Muscle, 16: e13806, 10.1002/jcsm.13806.40254030 PMC12009637

In the “Results” section of the Abstract, the text: “Nine eligible RCTs with 1838 participants were included in this study” was incorrect. This should have read: “Nineteen eligible RCTs with 1838 participants were included in this study”. The correct number reflects the total studies included in our meta‐analysis (19, not 9). This does not affect the overall conclusions, data integrity, or reproducibility of the study.

We apologize for this error.

