# Supplementary material for: Effectiveness of Mind–Body Exercise in Older Adults With Sarcopenia and Frailty: A Systematic Review and Meta‐Analysis
Source: J Cachexia Sarcopenia Muscle. 2025 Apr 20;16(2):e13806. doi: 10.1002/jcsm.13806 (PMC12009637; doi:10.1002/jcsm.13806)
Supplement: Supplementary file 1 — Appendix Table S1 Search strategy. Appendix Table S2 GRADE assessments. Reference: Romina Brignardello‐Petersena, Reem A. Mustafa, Reed A.C. Siemieniuk, M. Hassan Murad, Thomas Agoritsasa, Ariel Izcovich, Holger J. Sch€unemann, Gordon H. Guyatta, for the GRADE Working Group. GRADE approach to rate the certainty from a network meta‐analysis: addressing incoherence. J Clin Epidemiol. 2019 Apr; 108:77–85. doi: 10.1016/j.jclinepi.2018.11.025. Epub 2018 Dec 5. Appendix Table S3 Results of subgroup analysis based on exercise prescriptions. CRT, Chair Rise Test; TUGT, Timed‐Up‐and‐Go Test. Figure S1 Forest plot for different exercise types on grip strength. Figure S2 Forest plot for different exercise frequency on grip strength. Figure S3 Forest plot for different exercise duration on grip strength. Figure S4 Forest plot for different exercise types on CRT..CRT, Chair Rise Test. Figure S5 Forest plot for different exercise frequency on CRT..CRT, Chair Rise Test. Figure S6 Forest plot for different exercise types on TUGT.TUGT, Timed Up and Go Test. Figure S7 Forest plot for different exercise duration on TUGT..TUGT, Timed Up and Go Test. Figure S8 Forest plot for different exercise frequency on gait speed. Figure S9 Forest plot for different exercise duration on gait speed. [file JCSM-16-e13806-s001.docx]

# Appendix Table S1: Search strategy

**Date: through Feb. 22, 2024**

**S1.1 PubMed N =** **79**

| Steps | Search terms | Results |
| --- | --- | --- |
| #1 | "sarcopenia"[MeSH Terms] OR "sarcopeni*"[Title/Abstract] OR "frail"[Title/Abstract] OR "frailty"[Title/Abstract] OR "prefrail"[Title/Abstract] OR "prefrailty"[Title/Abstract] OR "pre-frail"[Title/Abstract] OR "pre-frailty"[Title/Abstract] | 53,589 |
| #2 | "Tai ji"[MeSH Terms] OR "tai chi"[Title/Abstract] OR "chi tai"[Title/Abstract] OR "tai ji quan"[Title/Abstract] OR "ji quan tai"[Title/Abstract] OR "quan tai ji"[Title/Abstract] OR "Taiji"[Title/Abstract] OR "Taijiquan"[Title/Abstract] OR "t'ai chi"[Title/Abstract] OR "tai chi chuan"[Title/Abstract] OR  "qigong"[MeSH Terms] OR "qi gong"[Title/Abstract] OR "chi gung"[Title/Abstract] OR "chi kung"[Title/Abstract] OR "baduanjin"[Title/Abstract] OR "yijinjing"[Title/Abstract] OR "liuzijue"[Title/Abstract] OR "wuqinxi"[Title/Abstract] OR  "yoga"[MeSH Terms] OR "yog*"[Title/Abstract] OR "pranayama"[Title/Abstract] OR "asana*"[Title/Abstract] OR "dhyana"[Title/Abstract] OR "pilates"[Title/Abstract] OR  "traditional Chinese exercise"[Title/Abstract] OR "Chinese traditional exercise"[Title/Abstract] OR "mind body"[Title/Abstract] OR "mind exercise"[Title/Abstract] | 11,841 |
| #3 | "muscle strength"[MeSH Terms] OR "muscle mass"[Title/Abstract] OR "muscle index"[Title/Abstract] OR "muscle size"[Title/Abstract] OR "muscle thickness"[Title/Abstract] OR "fat free mass"[Title/Abstract] OR "lean mass"[Title/Abstract] OR "muscle strength"[Title/Abstract] OR "muscular strength"[Title/Abstract] OR "muscle power"[Title/Abstract] OR "hand strength"[MeSH Terms] OR "handgrip strength"[Title/Abstract] OR "grip strength"[Title/Abstract] | 116,303 |
| #4 | ("musc*"[Title/Abstract] AND ("function*"[Title/Abstract] OR "size"[Title/Abstract] OR "volume"[Title/Abstract] OR "enlarge*"[Title/Abstract])) OR ("physical"[Title/Abstract] AND ("fitness"[Title/Abstract] OR "function*"[Title/Abstract] OR "capacity"[Title/Abstract] OR "disability"[Title/Abstract] OR "perform*"[Title/Abstract])) | 647,226 |
| #5 | #3 OR #4 | 715,083 |
| #6 | #1 AND #2 AND #5 | 79 |

**S1.2 Embase N = 121**

| Steps | Search terms | Results |
| --- | --- | --- |
| #1 | 'Sarcopenia'/exp OR 'sarcopeni*':ab,ti OR 'frail':ab,ti OR 'frailty':ab,ti OR 'prefrail':ab,ti OR 'prefrailty':ab,ti OR 'pre-frail':ab,ti OR 'pre-frailty':ab,ti | 79327 |
| #2 | 'tai ji'/exp OR 'tai chi':ab,ti OR 'chi tai':ab,ti OR 'tai ji quan':ab,ti OR 'ji quan tai':ab,ti OR 'quan tai ji':ab,ti OR 'taijiquan':ab,ti OR 'tai chi chuan':ab,ti OR 'taiji':ab,ti OR 'qigong'/exp OR 'qi gong':ab,ti OR 'chi gung':ab,ti OR 'chi kung':ab,ti OR 'baduanjin':ab,ti OR 'yijinjing':ab,ti OR 'liuzijue':ab,ti OR 'wuqinxi':ab,ti OR 'yoga'/exp OR 'yog*':ab,ti OR 'pranayama':ab,ti OR 'asana*':ab,ti OR 'dhyana':ab,ti OR 'pilates':ab,ti OR 'traditional Chinese exercise':ab,ti OR 'Chinese traditional exercise':ab,ti OR 'mind body':ab,ti OR 'mind exercise':ab,ti | 33142 |
| #3 | 'muscle strength'/exp OR 'muscle mass':ab,ti OR 'muscle index':ab,ti OR 'muscle size':ab,ti OR 'muscle thickness':ab,ti OR 'fat free mass':ab,ti OR 'lean mass':ab,ti OR 'muscle strength':ab,ti OR 'muscular strength':ab,ti OR 'muscle power':ab,ti OR 'hand strength'/exp OR 'handgrip strength':ab,ti OR 'grip strength':ab,ti OR 'musc* function*':ab,ti OR 'musc* size':ab,ti OR 'musc* volume':ab,ti OR 'musc* enlarge*':ab,ti OR 'physical fitness'/exp OR 'physical function*':ab,ti OR 'physical capacity':ab,ti OR 'physical disability':ab,ti OR 'physical perform*':ab,ti | 313663 |
| #4 | #1 AND #2 AND #3 | 121 |

**S1.3 Cochrane N = 150**

| Steps | Search terms | Results |
| --- | --- | --- |
| #1 | MeSH descriptor: [Sarcopenia] explode all trees | 869 |
| #2 | (sarcopeni*):ti,ab,kw OR (frail):ti,ab,kw OR (frailty):ti,ab,kw OR (prefrail):ti,ab,kw OR (prefrailty):ti,ab,kw OR (pre-frail):ti,ab,kw OR (pre-frailty):ti,ab,kw | 7,566 |
| #3 | #1 OR #2 | 7566 |
| #4 | (tai chi):ti,ab,kw OR (chi tai):ti,ab,kw OR (tai ji quan):ti,ab,kw OR (ji quan tai):ti,ab,kw OR (quan tai ji):ti,ab,kw OR (Taiji):ti,ab,kw OR (Taijiquan):ti,ab,kw OR (t'ai chi):ti,ab,kw OR (tai chi chuan):ti,ab,kw OR (qi gong):ti,ab,kw OR (chi gung):ti,ab,kw OR (chi kung):ti,ab,kw OR (baduanjin):ti,ab,kw OR (yijinjing):ti,ab,kw OR (liuzijue):ti,ab,kw OR (wuqinxi):ti,ab,kw OR (yog*):ti,ab,kw OR (pranayama):ti,ab,kw OR (asana*):ti,ab,kw OR (dhyana):ti,ab,kw OR (pilates):ti,ab,kw OR (traditional Chinese exercise):ti,ab,kw OR (Chinese traditional exercise):ti,ab,kw OR (mind body):ti,ab,kw OR (mind exercise):ti,ab,kw | 13,677 |
| #5 | MeSH descriptor: [Tai Ji] explode all trees | 500 |
| #6 | MeSH descriptor: [Qigong] explode all trees | 148 |
| #7 | MeSH descriptor: [Yoga] explode all trees | 1,232 |
| #8 | #4 OR #5 OR #6 OR #7 | 13,744 |
| #9 | (muscle mass):ti,ab,kw OR (muscle index):ti,ab,kw OR (muscle size):ti,ab,kw OR (muscle thickness):ti,ab,kw OR (fat free mass):ti,ab,kw OR (lean mass):ti,ab,kw OR (muscle strength):ti,ab,kw OR (muscular strength):ti,ab,kw OR (muscle power):ti,ab,kw OR (handgrip strength):ti,ab,kw OR (grip strength):ti,ab,kw OR (musc* function*):ti,ab,kw OR (musc* size):ti,ab,kw OR (musc* volume):ti,ab,kw OR (musc* enlarge*):ti,ab,kw OR (physical fitness):ti,ab,kw OR (physical function*):ti,ab,kw OR (physical capacity):ti,ab,kw OR (physical disability):ti,ab,kw OR (physical perform*):ti,ab,kw | 155,503 |
| #10 | MeSH descriptor: [Muscle Strength] explode all trees | 8,775 |
| #11 | MeSH descriptor: [Hand Strength] explode all trees | 2,160 |
| #12 | #9 OR #10 OR #11 | 155,697 |
| #13 | #3 AND #8 AND 12 | 150 |

**S1.4 Supplement table 4- Web of Science N = 216**

| Steps | Search terms | Results |
| --- | --- | --- |
| #1 | TS=("sarcopenia" OR "sarcopeni*" OR "frail" OR "frailty" OR "prefrail" OR "prefrailty" OR "pre-frail" OR "pre-frailty") | 102,948 |
| #2 | TS=("Tai ji" OR "tai chi" OR "chi tai" OR "tai ji quan" OR "ji quan tai" OR "quan tai ji" OR "Taiji" OR "Taijiquan" OR "t'ai chi" OR "tai chi chuan" OR"qigong" OR "qi gong" OR "chi gung" OR "chi kung" OR "baduanjin" OR "yijinjing" OR "liuzijue" OR "wuqinxi" OR "yoga" OR "yog*" OR "pranayama" OR "asana*"OR "dhyana" OR "pilates" OR "traditional Chinese exercise" OR "Chinese traditional exercise" OR "mind body" ) | 99,416 |
| #3 | TS=("muscle mass"OR "muscle index" OR "muscle size" OR "muscle thickness" OR "fat free mass" OR "lean mass" OR "muscle strength" OR "muscle strength" OR "muscular strength" OR "muscle power" OR "hand strength" OR "handgrip strength" OR "grip strength") | 189,463 |
| #4 | TS= ("musc* function*" OR "musc* size" OR "musc* volume" OR "musc* enlarge*" OR "physical fitness" OR "physical function*" OR "physical capacity" OR "physical disability" OR "physical perform*") | 212,754 |
| #5 | #3 OR #4 | 364,548 |
| #6 | #1 AND #2 AND #5 | 216 |

**S1.5 Supplement table 5 PsycInfo N = 15**

| Steps | Search terms | Results |
| --- | --- | --- |
| S1 | MH sarcopenia OR sarcopeni* OR frail OR frailty OR prefrail OR prefrailty OR pre-frail OR pre-frailty | 8,473 |
| S2 | MH "Tai ji" OR AB "tai chi" OR AB "chi tai" OR AB "tai ji quan" OR AB "ji quan tai" OR AB "quan tai ji" OR AB "Taiji" OR AB "Taijiquan" OR AB "t'ai chi" OR AB "tai chi chuan" OR MH "qi gong" OR AB "chi gung" ORAB "chi kung" OR AB "baduanjin" OR AB "yijinjing" OR AB "liuzijue" OR AB "wuqinxi" OR MH yoga OR AB yog* OR AB pranayama OR AB asana* OR AB dhyana OR AB pilates OR AB "traditional Chinese exercise" OR AB "Chinese traditional exercise" OR AB "mind body" OR AB "mind exercise" | 9,362 |
| S3 | MH "muscle strength" OR AB "muscle mass" OR AB "muscle index" OR AB "muscle size" OR AB "muscle thickness" OR AB "fat free mass" OR AB "lean mass" OR AB "muscle strength" OR AB "muscular strength" OR AB "muscle power" OR MH "hand strength" OR AB "handgrip strength" OR AB "grip strength" OR AB "musc* function*" OR AB "musc* size" OR AB "musc* volume" OR AB "musc* enlarge*" OR AB "physical fitness" OR AB "physical function*" OR AB "physical capacity" OR AB "physical disability" OR AB "physical perform*" | 20,490 |
| S4 | S1 AND S2 AND S3 | 15 |

**S1.6 Supplement table 6-** **CINAHL N = 48**

| Steps | Search terms | Results |
| --- | --- | --- |
| S1 | MH sarcopenia OR sarcopeni* OR frail OR frailty OR prefrail OR prefrailty OR pre-frail OR pre-frailty | 28,814 |
| S2 | MH "Tai ji" OR AB "tai chi" OR AB "chi tai" OR AB "tai ji quan" OR AB "ji quan tai" OR AB "quan tai ji" OR AB "Taiji" OR AB "Taijiquan" OR AB "t'ai chi" OR AB "tai chi chuan" OR MH "qi gong" OR AB "chi gung" ORAB "chi kung" OR AB "baduanjin" OR AB "yijinjing" OR AB "liuzijue" OR AB "wuqinxi" OR MH yoga OR AB yog* OR AB pranayama OR AB asana* OR AB dhyana OR AB pilates OR AB "traditional Chinese exercise"  OR AB "Chinese traditional exercise" OR AB "mind body" OR AB "mind exercise" | 18,085 |
| S3 | MH "muscle strength" OR AB "muscle mass" OR AB "muscle index" OR AB "muscle size" OR AB "muscle thickness" OR AB "fat free mass" OR AB "lean mass" OR AB "muscle strength" OR AB "muscular strength" OR AB "muscle power" OR MH "hand strength" OR AB "handgrip strength" OR AB "grip strength" OR AB "musc* function*" OR AB "musc* size" OR AB "musc* volume" OR AB "musc* enlarge*" OR AB "physical fitness" OR AB "physical function*" OR AB "physical capacity" OR AB "physical disability" OR AB "physical perform*" | 71,532 |
| S4 | S1 AND S2 AND S3 | 48 |

**S1.7 CNKI中国知网 N = 36**

((主题=肌少症OR 篇名=肌少症 OR摘要=肌少症OR主题=肌肉减少症OR篇名=肌肉减少症OR摘要=肌肉减少症OR主题=骨骼肌减少症OR篇名=骨骼肌减少症OR摘要=骨骼肌减少症) AND (主题=身心运动 OR主题=身心疗法OR主题=太极 OR主题=八段锦 OR主题=气功 OR主题=瑜伽 OR主题=普拉提 OR 主题=五禽戏 OR主题=易筋经 OR 主题=六字诀))

条件：中英文扩展，时间不限 (模糊匹配)

**S1.8中国万方N = 345**

主题: (身心运动 OR 身心疗法 OR 太极 OR八段锦 OR 气功 OR 瑜伽 OR 易筋经 OR 五禽戏 OR 六字诀) AND 主题: (肌少症 OR 肌肉减少症 OR 骨骼肌减少症)

条件：中英文扩展，主题词扩展，时间不限 (模糊匹配)

**S1.9 维普N = 29**

(((((题名或关键词=骨骼肌减少症 OR 题名或关键词=sarcopenia) OR 题名或关键词=肌肉减少症) OR 题名或关键词=少肌症) OR 题名或关键词=肌少症) AND ((((((((题名或关键词=身心运动 OR 题名或关键词=身心疗法) OR 题名或关键词=太极) OR 题名或关键词=八段锦) OR 题名或关键词=瑜伽) OR 题名或关键词=普拉提) OR 题名或关键词=易筋经) OR 题名或关键词=气功) OR 题名或关键词=六字诀))

# Appendix Table S2: GRADE assessments

Reference:

Romina Brignardello-Petersena, Reem A. Mustafa, Reed A.C. Siemieniuk, M. Hassan Murad, Thomas Agoritsasa, Ariel Izcovich, Holger J. Sch€unemann, Gordon H. Guyatta, for the GRADE Working Group. GRADE approach to rate the certainty from a network meta-analysis: addressing incoherence. J Clin Epidemiol. 2019 Apr; 108:77-85. doi: 10.1016/j.jclinepi.2018.11.025. Epub 2018 Dec 5.

| **MBE Compared to Passive Control for Sarcopenia and Frailty** | | | | | | |
| --- | --- | --- | --- | --- | --- | --- |
| **Patient or population:** Patients with sarcopenia and frailty **Settings:** Clinic **Intervention:** MBE **Comparison:** Passive control | | | | | | |
| **Outcomes** | **Illustrative comparative risks* (95% CI)** | | **Relative effect (95% CI)** | **No of Participants (studies)** | **Quality of the evidence (GRADE)** | **Comments** |
|  | Assumed risk | Corresponding risk |  |  |  |  |
|  | **Passive control** | **MBE** |  |  |  |  |
| **Muscle mass** Muscle mass |  | The mean muscle mass in the intervention groups was **0.33 lower** (2.39 lower to 1.72 higher) |  | 206 (4 studies) | ⊕⊕⊝⊝ **low**^1^ | WMD -0.09 (-0.52 to  0.34) |
| **Muscle strength** Grip strength |  | The mean muscle strength in the intervention groups was **0.97 higher** (0.07 to 1.88 higher) |  | 775 (11 studies) | ⊕⊕⊕⊝ **moderate**^2^ | WMD 0.97 (0.07 to  1.88) |
| **TUGT** Timed Up-and-Go Test |  | The mean TUGT in the intervention groups was **4.04 lower** (5.38 to 2.71 lower) |  | 266 (5 studies) | ⊕⊕⊕⊝ **moderate**^3^ | WMD -4.04 (-5.38 to  -2.71) |
| **GS** Gait speed |  | The mean GS in the intervention groups was **0.02 higher** (0.01 lower to 0.05 higher) |  | 880 (7 studies) | ⊕⊝⊝⊝ **very low**^4^ | WMD 0.02 (-0.01 to  0.05) |
| **CRT** Chair Rise Test |  | The mean CRT in the intervention groups was **1.39 higher** (2.12 lower to 4.9 higher) |  | 505 (8 studies) | ⊕⊝⊝⊝ **very low**^5^ | SMD 0.46 (-0.47 to  1.40) |
| **BBS** Berg Balance Scale |  | The mean BBS in the intervention groups was **3.63 higher** (0.38 to 6.87 higher) |  | 73 (3 studies) | ⊕⊕⊕⊝ **moderate**^6^ | WMD 3.63 (0.38 to  6.87) |
| **6mWT**  The 6m Walk Test |  | The mean 6mWT in the intervention groups was **45.33 higher** (47.79 lower to 138.45 higher) |  | 120 (3 studies) | ⊕⊝⊝⊝ **very low**^7^ | WMD 45.33 (-47.79 to 138.45) |
| *The basis for the **assumed risk** (e.g. the median control group risk across studies) is provided in footnotes. The **corresponding risk** (and its 95% confidence interval) is based on the assumed risk in the comparison group and the **relative effect** of the intervention (and its 95% CI). **CI:** Confidence interval; | | | | | | |
| GRADE Working Group grades of evidence **High quality:** Further research is very unlikely to change our confidence in the estimate of effect.  **Moderate quality:** Further research is likely to have an important impact on our confidence in the estimate of effect and may change the estimate. **Low quality:** Further research is very likely to have an important impact on our confidence in the estimate of effect and is likely to change the estimate. **Very low quality:** We are very uncertain about the estimate. | | | | | | |
| ^1^ Downgraded once for inconsistency due to heterogeneity and once for the number of comparisons was lower than 10 and it was not possible to exclude the presence of a publication bias ^2^ Downgraded once for study limitations due to high or unclear risk of bias. ^3^ Downgraded once for study limitations due to high or unclear risk of bias. ^4^ Downgraded once for study limitations due to high or unclear risk of bias, once for inconsistency due to heterogeneity and once for the number of comparisons was lower than 10 and it was not possible to exclude the presence of a publication bias. ^5^ Downgraded once for study limitations due to high or unclear risk of bias, once for inconsistency due to heterogeneity and once for the number of comparisons was lower than 10 and it was not possible to exclude the presence of a publication bias. ^6^ Downgraded once for study limitations due to high or unclear risk of bias. ^7^ Downgraded once for inconsistency due to heterogeneity, once for imprecision due to low participant numbers, and once for the asymmetrical distribution of the results in the funnel plot. | | | | | | |

# Appendix Table S3: Results of subgroup analysis based on exercise prescriptions

| **Categories** | **Outcomes measures** | **Subgroup** | **Studies**  **(participants)** | **SMD/WMD**  **(95%CI)** | **Heterogeneity**  **(I^2^, *P*-value)** |
| --- | --- | --- | --- | --- | --- |
|  | **Exercise types** | | | | |
| **Muscle function** | Grip strength | Tai Chi | 4 (251) | 0.53 (-0.88,1.94) | I^2^ = 0%, *P* = 0.46 |
|  |  | Yijinjing | 6 (484) | 1.27 (0.04, 2.51) | I^2^ = 41%, *P* = 0.04 |
|  |  | Baduanjin | 1 (40) | 1.42 (-2.65, 5.49) | *P* = 0.49 |
| **Physical performance** | CRT | Tai Chi | 2 (130) | 1.41 (0.18, 2.63) | I^2^ = 90%, *P* = 0.02 |
|  |  | Yijinjing | 5 (335) | -0.00 (-1.46, 1.46) | I^2^ = 97%, *P* = 1.00 |
|  |  | Baduanjin | 1(40) | 0.75 (0.10, 1.39) | *P* = 0.02 |
|  | TUGT | Yijinjing | 4 (226) | -4.32 (-6.29, -2.35) | I^2^ = 32%, *P* < 0.001 |
|  |  | Baduanjin | 1 (40) | -3.81 (-5.63, -1.99) | *P* < 0.001 |
|  | **Exercise frequency** | | | | |
| **Muscle function** | Grip strength | Low | 4 (347) | 0.31(-0.91, 1.53) | I^2^ = 0%, *P* = 0.62 |
|  |  | Moderate | 1 (71) | 0.56 (-2.11, 3.23) | *P* = 0.68 |
|  |  | High | 6 (392) | 2.02 (0.50, 3.54) | I^2^ = 23%, *P* = 0.009 |
| **Physical performance** | CRT | Moderate | 4 (276) | 1.32 (0.70, 1.94) | I^2^ = 82%, *P* < 0.001 |
|  |  | High | 4 (229) | -0.47 (-2.38, 1.45) | I^2^ = 97%, *P* = 0.63 |
|  | Gait speed | Low | 5 (750) | 0.01 (-0.02, 0.03) | I^2^ = 87%, *P* = 0.47 |
|  |  | Moderate | 2 (130) | -0.62 (-1.01, -0.23) | I^2^ = 24%, *P* = 0.002 |
| **Exercise duration** | | | | | |
| **Muscle function** | Grip strength | Short | 8 (575) | 1.35 (0.10, 2.59) | I^2^ = 18%, *P* = 0.03 |
|  |  | Medium | 2 (150) | 41.61 (5.94, 77.28) | I^2^ = 100%, *P* = 0.02 |
|  |  | Long | 1 (44) | -1.00 (-4.03, 2.03) | *P* = 0.52 |
| **Physical performance** | TUGT | Short | 4 (230) | -3.55 (-5.02, -2.08) | I^2^ = 0%, *P* < 0.001 |
|  |  | Medium | 1 (36) | -6.40 (-9.60, -3.20) | *P* < 0.001 |
|  | Gait speed | Short | 2 (130) | -0.62 (-1.01, -0.23) | I^2^ = 24%, *P* = 0.002 |
|  |  | Medium | 2 (288) | 0.02 (0.01, 0.03) | I^2^ = 0%, *P* < 0.001 |
|  |  | Long | 3 (462) | 0.01 (-0.04, 0.05) | I^2^ = 92%, *P* = 0.77 |

CRT**,** Chair Rise Test; TUGT**,** Timed-Up-and-Go Test
